# A professional nurse’s understanding of quality nursing care in Limpopo province, South Africa

**DOI:** 10.4102/curationis.v46i1.2322

**Published:** 2023-03-10

**Authors:** Maggie Nyelisani, Lufuno Makhado, Takalani Luhalima

**Affiliations:** 1 Department of Advanced Nursing Science, Faculty of Health Sciences, University of Venda, Thohoyandou, South Africa; 2 Department of Public Health, Faculty of Health Sciences, University of Venda, Thohoyandou, South Africa

**Keywords:** professional nurse, quality nursing care, hospital, teamwork, patients’ needs

## Abstract

**Background:**

Quality has increasingly become a critical part of life in every aspect. Patients are today continuously looking for good quality services from health professionals. Professional nurses are expected to render quality care to fulfil the patients’ healthcare needs. Poor nursing care has led to several litigations and the loss of patients’ lives. It is essential to explore professional nurses’ viewpoints regarding quality nursing care.

**Objectives:**

To explore and describe the understanding of professional nurses regarding quality care rendered to patients in the selected hospitals of Limpopo Province.

**Method:**

This study utilised a qualitative, exploratory-descriptive design. Individual semi-structured interviews were conducted for data collection. Participants comprised 35 professional nurses who were purposely selected. Data collected were audio-recorded and transcribed verbatim. Data were analysed using Tech’s eight-step data coding process, which led to the emergence of themes and sub-themes. Trustworthiness was ensured through credibility, confirmability, dependability, and transferability.

**Results:**

Three themes emerged: professional nurses’ descriptions, meanings, and expectations of quality nursing care. The findings highlight that quality nursing care means meeting patients’ needs through advocacy, empathy, fulfilment of patients’ needs, good interpersonal relationships and teamwork. Challenges experienced included the lack of resources and staff shortage.

**Conclusion:**

Hospital management needs to develop effective ways to support professional nurses in delivering quality nursing care. In discussion with the Department of Health (DoH), hospitals should be fully equipped with resources to render quality care to patients. Evaluation of service quality and patient satisfaction should be ongoing for improving the quality of patient care.

**Contribution:**

The study reveals that professional nurses perceive quality nursing care differently. Moreover, it emphasises the importance of maintaining and promoting quality nursing care as the cornerstone of healthcare.

## Introduction

The World Health Organization (WHO) states that quality healthcare must be rendered to individuals and society (WHO 2013). Caring expressed in nursing is the intended and actual presence of the nurse who is recognised as a person who is loving, caring and growing in caring. Caring involves tending to the patient’s needs, providing physical and spiritual well-being, and building trust (Franjić 2018; Karlsson & Pennbrant 2020).

Quality nursing care is viewed differently by healthcare professionals and patients. Therefore, competent nursing care is considered quality nursing care by healthcare professionals. Good interpersonal relationships and caring are perceived as quality nursing care. According to Hartley et al. (2020) and Treinen (2021), nursing relationships with patients have been seen as a major therapeutic interpersonal process that cooperates with other human processes to enable individuals and communities to be healthy. This indicates that the nurse–patient relationship ensures that a patient’s needs are attended to as the patient will be free to voice their concerns, and it reduces the days of hospital stay and improves the quality of life. Relationships with family members make it possible to render quality care.

According to West et al. (2021), health care providers discussed many obstacles to providing quality healthcare in South Africa, which falls under the foundational categories of workforce and tools. The lack of human and material resources affected all aspects of care, which can be traced back to a lack of human resources. It has previously been documented that in South Africa, there is understaffing, a lack of training, poor infrastructure, a lack of clinical care space, and the challenges associated with providing care because of these deficiencies.

A study conducted by Stalpers et al. (2017) in the Netherlands found that nurses’ perceived quality of care and job satisfaction contributed to quality outcomes for patients and nurses. Nursing care quality can be improved by understanding specific factors influencing nurses’ perceived quality. A positive correlation was found between nurse-perceived quality and work environment characteristics, adequate staffing, patient-centredness, competent colleagues, and educational support.

Chakraborty, Kaynak and Pagán (2021) in their study on bridging hospital quality leadership to patient care quality commented that an understanding of what initiates quality in the delivery of healthcare services is an important patient care experience. They further commented that hospitals could provide high-quality patient care if their leaders focus on effectively integrating all available hospital technologies, and building and sustaining effective healthcare teams could reduce medical errors.

The lack of equipment, inadequate training, and limited infrastructure in Limpopo Province make it difficult to provide optimal care to critically ill patients (Netshisaulu, Malelelo-Ndou & Ramathuba 2019). Apart from providing suboptimal patient care, healthcare professionals face challenges relating to non-adherence to protocols and instructions and the challenge of operating beyond their scope of practice. Intensive patient care was suboptimal because of lack of resources. Patients could contract infections, which compromised their safety and the quality of care they receive. Quality care to patients seems to be compromised in healthcare institutions, as evidenced by the above mentioned studies.

## Problem statement

Professional nurses are expected to render quality care to fulfil patients’ healthcare needs in their hospitals. Poor healthcare has led to several litigations and the loss of patients’ lives. This has become a problem in South Africa. Currently, the Department of Health’s claims have increased by 132% in South Africa. The Department had paid a claim amounting R17 million in 1 year to a member, and this is unduly costly for the Department of Health (Bateman 2011). It was against this background that this study aimed to explore the experiences of professional nurses’ understanding of quality nursing care in the public hospitals of Limpopo Province.

## Aim

This qualitative descriptive study aimed to explore and describe the professional nurses’ understanding of quality nursing care at selected public hospitals in Limpopo Province.

## Research methods and design

### Research design

This study utilised a qualitative, exploratory-descriptive design approach to explore and describe professional nurses’ understanding of quality nursing care in the selected public hospitals. This design was suitable for this study as it provided professional nurses with an opportunity to describe their views or perspectives regarding quality nursing care.

### Study context

This study was conducted in selected public hospitals in Limpopo province. The selected hospitals are situated in Vhembe, Capricorn and Mopani districts. The settings were selected because they are the major public hospitals in the mentioned districts, provide general patient care, and have a high number and different categories of professional nurses working in the hospital settings.

### Population and sampling

The target population comprised professional nurses working in selected hospitals in Limpopo Province. A purposive sample of 35 professional nurses with at least five years of experience working in these public hospitals was selected. Data saturation was reached at participant number 30, and an additional five participants were included to ascertain if no new information emerged.

### Data collection

The data were collected using in-depth, face-to-face interviews. It took approximately 30–45 min to conduct the audio-recorded interviews with the participants’ permission. A semi-structured interview (SSI) guide was used as a tool, which consisted of a pre-determined set of open-ended questions (questions that prompted discussion). This provided an opportunity for the interviewer to explore particular responses from participants. The procedure followed during data collection is that the professional nurses were contacted by their District Executive Managers (DEM), Chief Executive Officers (CEO), and Nurse Managers in the hospitals. Permissions were sought after the researcher had applied to the DEM and hospitals’ CEO telephonically and in writing to conduct the study and collect data. Managers arranged the venue and selected professional nurses according to the study requirements. Interviews took place mostly on Wednesdays as the staff were on a double shift, meaning that extra personnel were on duty as it was a changeover of shifts.

### Data analysis

The researchers and an independent co-coder used Tesch’s eight-step method of data analysis to analyse the data (Creswell 2014). The steps for this study’s data analysis included the transcription of raw data verbatim, perusing it, and carefully reading the transcripts to have a good understanding of the data while reflecting on their meanings. This was followed by the removal of unrelated details, the preparation for data coding, and properly developing and uniting the related concepts and categories into themes for clarity and validation. This was facilitated to ensure that the collected data reflected the participants’ authentic ideas (Creswell 2014).

### Measures to ensure trustworthiness

Trustworthiness in this study was ensured through maintaining credibility, confirmability, transferability, and dependability, as summarised by Creswell (2014). To ensure credibility of the study findings, the researcher interacted with participants to get hold of their true viewpoints. Prolonged engagement was ensured by spending sufficient time with participants. Member checking was done by involving the supervisor and co-supervisor in verifying the researchers’ interpretations. Data analysis was done with an independent co-coder to ensure the study’s validity (Polit & Beck 2017). Data collection and analysis were conducted with objectivity to ensure confirmability (Creswell 2014). To confirm the audit trail, transcripts, tape-recorded information and field notes were made available by researchers. An independent coder was used to check the data collected, and promoters of the study were involved as independent checkers throughout the research process. Transferability was ensured through purposive sampling of participants, data collection using an interview guide and a thick description of findings presented (Polit & Beck 2017). Discussion of information from participants was done, and findings were made available with quotes. Transparency was fostered by clearly presenting the research methodology and findings, thus ensuring dependability.

### Ethical considerations

Ethical clearance for this study was attained from the University of Venda Human and Clinical Trial Research Ethics Committee (HCTREC) (SHS/20/PDC/17/1206). The Limpopo provincial Department of Health granted permission to conduct the study. At the same time, the District Executive Managers (DEM), Chief Executive Officers (CEO) and Nurse Managers in the hospitals had also granted further permission. Informed written consent was obtained from participants who knew that anonymity and confidentiality would be maintained throughout the study. The names of the participants would not appear on documentation but their code numbers. Participation in the study was voluntary.

## Results

### Demographic characteristics of participants

Demographic characteristics of the study comprised 35 professional nurses from the selected public hospitals of Limpopo Province. Majority of participants were females (*n* = 31; 88.6%) and were males (*n* = 4; 11.4%). Participants’ qualifications were Diploma in Nursing (*n* = 23; 65.7%) and Bachelor in Nursing including further qualifications (*n* = 12; 34.3%). Table 1 outlines the characteristics of participants.

**TABLE 1:** Characteristics of participants.

Item	Biographical data	Count (*n*)	%
Gender	Male	4	11.4
	Female	31	88.6
Age	31–34 years	8	22.8
	35–50 years	16	45.7
	Above 51 years	11	31.4
Qualification	Diploma in Nursing	23	65.7
	Bachelor in Nursing or further qualifications	12	34.3
Years of experience	> 5	8	22.8
	10–20	14	40.0
	21–30	8	22.8
	> 30	5	14.2

The findings are on the data collected through individual SSI conducted from the 35 participants on the experiences of professional nurses regarding their experiences in the understanding of quality care of patients in the selected hospitals. Understanding the nature of quality care to be rendered to patients is an essential aspect of nursing. The acquired knowledge by professional nurses enables them to share the following experiences: professional nurses’ description of quality care, professional nurses’ meaning of quality care and professional nurses’ expectations related to them receiving quality nursing care. The three themes that emerged are described in narrative form as experienced by professional nurses concerning quality nursing care. The participant identifiers allocated were hospital (*H* and number), participant (*P* number) and gender (female = *F*). Table 2 outlines the identified themes and sub-themes.

**TABLE 2:** An outline of the identified themes and sub-themes.

Themes	Sub-themes
1. Professional nurses’ description of quality nursing care	Advocacy for patients
	Relationship with the patients
	Relationship with the family
	Total patient care
	Good working relationships among nurses
	Application of Batho-Pele principles
	Proper use of resources
	Use of guidelines and policies
2. Professional nurses’ meaning of quality nursing care	Fulfilment of needs
	Empathy
	Equal treatment of patients
	Do to others as I will want them to do to me
3. Professional nurses’ expectations related to patients receiving quality nursing care	Nurses to have positive attitudes
	Good nurse–patient relationship
	Teamwork
	Lack of specialisation

### Theme 1: Professional nurses’ description of quality nursing care

Theme 1 outlines the professional nurses’ own description of quality nursing care. It is composed of the sub-themes: advocacy for patients, relationship with the patients, relationship with the family, total patient care, good working relationship among nurses, application of Batho-Pele principles, proper use of resources, and use of guidelines and policies. The discussion of sub-themes is as follows:

#### Subtheme 1.1: Advocacy for patients

Participants stated that advocacy for the patient is essential to quality care as professionals. During patient care, professional nurses advocate by speaking on behalf of the patient to meet their needs. This is reflected in the following comments:

‘I have to advocate for the patient; during the assessment, if you pick up abnormalities, you have to liaise with the doctor, and if the doctor prescribes the wrong medication, you have to speak for the patient.’ (H3, P3, F)

‘You tell them the time when a doctor is coming so that they can come and see the doctor. You advocate for the patient with problems, speak to the doctor, allow the patient to verbalize in each and everything she has.’ (H2, P1, F)

#### Subtheme 1.2: Relationship with the patients

Participants commented that maintaining good relationships renders quality care to patients. They further commented that one has to greet the patient, carry out doctors’ orders, and answer their questions. Participants verbalised the following statements:

‘Quality nursing care, according to my understanding, is to nurse the patient with high standard care, giving them a smile, when they ask you a question you answer them.’ (H2, P1, F)

‘The first impression to the patient where the healing of a patient comes, greeting patient first, second, carry out orders for the patient from the doctor and advocate for them.’ (H5, P3, F)

‘Some patients come to the hospital anxious. We must relieve anxiety, reassure the patient, make a relationship with patient …nurse/patient relationship should be good so that the patient can cope with the situation.’ (H2, P3, F)

#### Subtheme 1.3: Relationship with the family

Participants commented that quality care also involves having a good relationship with the patients’ family, involving them in their care, and informing them about their condition. Participants indicated this in the following manner:

‘When relatives come, if they ask what the problem with the patient is, tell them I’m a sister. I’m not allowed to tell you about the problem of the patient, the doctor will handle it.’ (H2, P3, F)

‘It means when I’m done with the patient care, every aspect has been touched, like in ICU. I’m ICU trained specialist. We work as a multidisciplinary team, which means everybody concerned must do his work and consider family, update family concerning the patient’s condition.’ (H3, P4, F)

#### Subtheme 1.4: Total patient care

Participants view total patient care as giving the patient care in totality, giving care to their physical aspect and treating them holistically until healing occurs. Participants highlighted this as follows:

‘This is nursing the patient in totality, like … meeting the needs of the patients ehh … mentally, physically, emotionally until the patient is healed with the medication.’ (H1, P4, F)

‘Nursing a patient physically, spiritually and emotionally, spending time with patient listening to the patient, be an advocate for the patient.’ (H5, P3, F)

#### Subtheme 1.5: Good working relationship among nurses

Professional nurses’ good working relationship was viewed as an essential aspect of quality care as the involvement of all inpatient care, including the patient themself, results in quality care. The following comments were given:

‘In rendering quality care … as nurses, we work hand in hand [*laughs*]. To get quality nursing care, we have to work as a team support each other because we are here for a common goal … to nurse the patient to get well and go …’ (H4, P1, F)

‘Participation is to give quality care to patient needs. It means all of us need to be involved, and teamwork is important, and the patient needs to be involved in his care.’ (H5, P4, F)

#### Subtheme 1.6: Application of Batho-Pele principles

Participants viewed the application of the Batho-Pele principles as part of rendering quality care to patients as patients rely on professional nurses for care, and the care that nurses render should be transparent and open. The following statements support the view of the participants:

‘Since I am a nurse, it means a lot to me because I would always like to provide is quality care to patients as patients rely on us [*yes* …] I think the rights of patients should be respected at all times, and Batho-Pele principles should be applied.’ (H5, P1, F)

‘Quality patient care is the care that is offered to the patient, providing patient with treatment, application of Batho-Pele Principles, taking care of patients.’ (H5, P5, F)

‘Patient has value for money-they pay for care rendered. Openness and transparency where patients should be given the information on their illness and their options to decide their healthcare interventions.’ (H5, P1, F)

#### Subtheme 1.7: Proper use of resources

Participants view proper use and optimal resources as aspects of giving quality care. The lack of such resources is viewed as preventing patients from receiving quality care. The following statements support the view of the participants:

‘Quality nursing patient care is providing service to patient using optimal resources at hand.’ (H4, P2, F)

‘Lack of resources and shortage of staff will prevent patients from receiving quality care.’ (H2, P2, F)

#### Subtheme 1.8: Use of guidelines and policies

Participants commented that as they give quality care to patients, they are guided by scientific guidelines and policies that work within a legal framework. They following comments by the participants present their views:

‘As a quality orientated person, you also need to look at adverse effects that can occur [sic] during nursing care. Again … you make sure that you stick to the set standards for the patient’s care, you follow the policy and regulations and the Act governing us, which is Act. no. 33 of 2005 …’ (H3, P2, F)

‘We are guided by steps, standards and protocols, e.g. In MDR patients. CPD is done in this way. People need to remind one another in this way.’ (H2, P5, F)

### Theme 2: Professional nurses’ meaning of quality nursing care

Theme 2 outlines the professional nurses’ own description of the meaning of quality nursing care. It is composed of the following sub-themes: fulfilment of patient needs, advocacy for patients, empathy, equal treatment of patients, and doing to others as I want them to do to me. The discussion of sub-themes follow.

#### Subtheme 2.1: Fulfilment of patients’ needs

Participants commented that quality care is the fulfilment of needs. Patients need their health needs to be fulfilled in time and referred to other multidisciplinary team members if required. They commented in the following statements:

‘It means all my needs should be fulfilled. If I am hungry, I should be fed; if I have a problem, let it be addressed and solved in time.’ (H1, P5, F)

‘I would expect the caregivers to treat me the right way. They should give me full care, if I need psychological care, they should refer me to get help …’ (H2, P5, F)

#### Subtheme 2.2: Empathy

Participants commented that empathy should be shown when nurses offer care to patients. The following comments were stated:

‘You nurse the patient in totality. You show empathy and advocate for the patient.’ (H2, P1, F)

#### Subtheme 2.3: Equal treatment for patients

Participants verbalised that all patients should receive equal treatment from professional healthcare providers, not considering who they are. Participants commented the following:

‘It means the manner in which you treat patients, and you should treat them equally. Apply the Batho-Pele principles. Patients should be treated the same in nursing.’ (H2, P5, F)

‘It means you must treat them in total, any culture, colour, sex, treat them the same, rich or poor.’ (H1, P7, F)

#### Subtheme 2.4: Do unto others as I want them to do to me

Participants, in the view of quality care rendered, reiterated that the quality of care they render to the patient is the same care they would like to receive. They stated the following:

‘It is the provision of service that I would like to give if I were in the patient’s same position or situation.’ (H4, P3, F)

‘It means what I want others to do to me is what I have to do to another person.’ (H1, P1, F)

### Theme 3: Professional nurses’ expectations related to patients receiving quality nursing care

Theme 3 outlines the professional nurses’ expectations related to patients receiving quality nursing care. It comprises the following sub-themes: nurses to have positive attitudes, good nurse–patient relationships, teamwork and the lack of specialisation. The discussion of sub-themes follow.

#### Subtheme 3.1: Nurses to have a positive attitude

Participants commented that nurses need to have good attitudes toward patients; this is an attribute they were taught during their training/development. This includes communicating well with patients. Another participant verbalised that patients should be treated well; patients lost their lives on account of being ill-treated by nurses rather than dying from the disease. They commented as follows:

‘We were developed we were told about our attitudes towards patients, how to communicate well with patients, and how we were brought up differently as nurses. If one of the nurses is having depression, we need to support them.’ (H2, P3, F)

‘I would expect to be treated well. I will expect a good attitude from the nurses … People die outside because of nurses’ attitudes. As a nurse, you need to treat patients with respect. If my nurses are rude to the patient, I reprimand them because I don’t want them to suffer. I’m living a good life because of what I give my patient.’ (H2, P1, F)

#### Subtheme 3.2: Good nurse–patient relationship

Participants commented that a good relationship between the nurse and the patient should exist. One participant reported that greeting a patient is the first impression where healing starts. Another participant verbalised that when nurses respond to patients’ calls for help, it is a way of rendering quality care. Participants highlighted that:

‘The first impression to the patient where the healing of a patient comes, greeting patient first, second, carry out orders for the patient from the doctor and advocate for them.’ (H5, P3, F)

‘When patients call you and are in need, you go and answer that bell. We can say you are providing quality nursing care to the patient. You don’t neglect them.’ (H1, P1, F)

#### Subtheme 3.3: Teamwork

Participants verbalised that teamwork is an essential aspect of quality care. They stated that patients should be involved in their care. They verbalised that although they work as a team, everyone has to do their work, and the family should also be involved. They commented as follows:

‘Participation is to give quality care patient needs. It means all of us to need to be involved, teamwork is important and patient needs to be involved in his care.’ (H5, P4, F)

‘It means when I’m done with the patient care, every aspect has been touched, like in ICU, I’m ICU trained specialist, we work as a team, means everybody concerned must do his work also considering family, update family concerning the condition of the patient.’ (H3, P4, F)

#### Subtheme 3.4: Lack of specialisation

Participants indicated that they wish more professional nurses should be trained as nurse specialists as these skills are needed in the hospital. Only a few professional nurses have these skills. They stated that these skills are essential as they will have the knowledge to teach others, including doctors. They commented as follows:

‘For professional nurses, I wish if they can train them for speciality, Trauma, ICU, Paeds etc. I myself [*sic*], I am an Occupational Health specialist, and I’m the only one in the hospital with this speciality.’ (H1, P1, F)

‘We need those specialists in the hospital. It’s important that we have them to be able to teach the doctors and nurses. We need them to teach us. We want to learn. We want them to be here in the hospital to teach us weekly, at least.’ (H1, P4, F)

## Discussion

Based on the findings of this study, it is conclusive that participants had their description of quality nursing care and the meaning thereof, including their expectations of them receiving quality care. A nurse as an advocate speaks on behalf of patients whose voices are not heard and is empathetic to the patient. The professional nurse acts on behalf of the patients before doctors and other members of the multidisciplinary team to ensure that the patients’ needs are met. In quality nursing care, values such as empathy and advocacy are essential aspects of quality care as professionals. Oliveira and Tariman (2017) highlighted that patient advocacy and empathy involve speaking on behalf of the patient and acting in the patient’s best interest. This implies that it is important that one has to be an advocate to speak on behalf of the patient so that their needs can be met continuously until they are discharged. The wellness and ability of the nurses as practitioners are vital. Self-worth and self-esteem are some of the factors that strengthen nurses; it enhances their skills and assists them in developing both mentally and physically (Jan et al. 2015). Nurses must maintain good interpersonal relationships between them and patients, including good relationships with patients’ families, thereby ensuring good quality care. This is supported by Hartley et al. (2020) and Zhou et al. (2018), and Treinen (2021), who stated that the nurses’ relationship with the patient and families has been seen as significant. This implies that a good nurse–patient relationship ensures that patients’ needs are attended to. Holistic nursing requires the nurse to provide care to patients based on a joint understanding of the patient’s physical, psychological, emotional, and spiritual aspects. Rajabpour and Rayyani (2019) stated that quality care meets physical needs by providing professional care, psychosocial support, satisfaction with care, and ensuring comprehensive care delivery to the patient. Therefore, this indicates that nurses need to be trained in holistic care skills. The Batho-Pele principles are essential in rendering quality care to patients (James & Miza 2015). Batho-Pele principles should be an inherent value of professional nurses; this includes the fact that patients need to receive equal treatment from nurses regardless of who they are. Batho-Pele principles ensure that patients are treated with respect. This is supported by Marron et al. (2020), that fairness and equality in the distribution of treatment among patients should reduce disparities. In addition, Ngidi and Dorasamy (2014) stated that effective, efficient and economical service provision can also be improved if Batho-Pele principles can be aligned to employment contracts and employees are assessed to ensure the effectiveness of Batho-Pele principles application.

Professional nurses’ teamwork and good attitudes towards patients are attributes that need to be inherent in them as they perform their duties. These are important aspects of quality care; this includes communicating well with patients. In supporting this view, Gholamzadeh et al. (2018) and Simko et al. (2017) commented that good attitudes between healthcare providers and patients are vital in achieving the goals of the care plan. This implies that good attitudes and teamwork enhance quality care. In addition, Lee (2011) stated that when managers facilitate professional peer attitudes and offer support effectively in the practice setting, these strongly enhance positive change. This confirms that when professional nurses are supported, they will be more productive and motivated to learn and work effectively to render quality patient care. Brekelmans, Poell and Van Wijk (2013) supported this by stating that employers and managers need to become involved by creating a positive working environment in institutions for employees. Documentation of nursing care rendered is an essential aspect of quality care. Kebede, Endris and Zegeye (2017) stated that most nursing care that nurses provide remains undocumented. The following factors were associated with nursing care documentation: nurse-to-patient ratio, in-service training, knowledge, and attitude of nurses toward nursing care documentation.

## Conclusion

Professional nurses must distinguish between their values and professional ethics as they nurse patients daily. Professional nurses have an essential function of becoming advocates to speak on behalf of the patient to meet their needs holistically. They need to treat patients equally without considering who they are. A good relationship between healthcare providers and patients is essential in achieving the goals of the care plan. Teamwork is a critical aspect of quality care. Everyone has to do their work so that medical errors are reduced. The Department of Health should ensure that hospitals are fully equipped with resources to render quality care to patients.

### Strength and limitations

This study shares valuable information on the quality nursing care of patients by professional nurses in healthcare settings. However, this study is limited to professional nurses in public hospitals. Hoping future research will consider the opinions of professional nurses from private hospitals and primary healthcare centres.

### Recommendations

Advocacy should be one of the inherent roles of professional nurses. Nurses should act with kindness when delivering care to patients. Training institutions should continuously reinforce values such as advocacy and empathy in ethics and legal knowledge in nursing care. Batho-Pele principles should be an inherent value of professional nurses and should be practised continuously to ensure that patients are treated with respect and consideration. Professional nurses should treat patients equally, irrespective of who they are. Nurses must have positive attitudes towards patient care if quality care is to be provided. Managers need to offer support by encouraging nurses to work as a team. Managers and the Department of Health should ensure that hospitals are fully equipped with resources to render quality care and ensure that scientific guidelines and policies are adhered to by staff.
